# Legal mobilization dilemmas in the Nordics – an autoethnographic reflection on the role of scholars in the asylum commission

**DOI:** 10.3389/fsoc.2024.1367517

**Published:** 2024-08-07

**Authors:** Anna Lundberg

**Affiliations:** Department of Sociology of Law, Faculty of Social Sciences, Lund University, Lund, Sweden

**Keywords:** legal mobilization, Sweden, migration law, scholarship of hope, autoethnography, activism

## Abstract

The present article investigates key dilemmas in collective legal mobilization initiatives in the field of asylum and migrants’ rights. Focusing on my own experiences from working in the Asylum Commission – a trans-sectional mobilization initiative that ran in Sweden from 2019 to 2022, involving researchers, civil society representatives, and professionals – I analyze two central dilemmas that characterized our work. First, I consider how we collectively struggled for the legal right to asylum and through this struggle also reproduced injustices and potential border control harms which are embedded in asylum regulations. Second, I analyze how the Commission strived to provide a knowledge-based account of the consequences of legislative changes post the long summer of migration in 2015 that would have an impact on future legislation, while simultaneously taking an open stand in solidarity with people who were excluded from the legislative process; i.e., asylum seekers. The article underlines the need for sociolegal research that highlights ways to address dilemmas in legal mobilization work and offers empirical insights from collective mobilization for migrants’ rights in a Northern European country.

## 1 Introduction

On June 19, 2019, around 30 people came together in a rented venue in the Swedish capital of Stockholm. We were activists in the refugee rights movement, including people with first-hand experience of going through the asylum procedure, professionals working with asylum seekers, and migration scholars. The group, which collectively possessed extensive knowledge of migration and asylum seekers’ living conditions in contemporary Sweden, had gathered to form a unique collaboration. We wanted to conduct a collective critical inquiry based on asylum seekers’ lived experiences and perspectives on migration law. Our focus was the time period following the Swedish government’s volte-face in asylum policy in November 2015.

Before the meeting in June, I had prepared, together with the other initiator of the Commission, an opinion piece titled “The Temporary Asylum Act - a major social experiment. We want to enable redress for those affected.” Sweden’s largest newspaper had agreed to publish our piece. We had also co-authored a draft document outlining the Commission’s planned work. Both these texts were scrutinized during the meeting.

While the project had no funding, the group was convinced that it must be carried out. Thousands of people residing in Sweden had been severely affected by hastily adopted new legislation and more restrictive legal provisions. Hundreds of undocumented people, asylum seekers, and people who had been notified of a deportation order had turned to civil society actors and local activist groups all over Sweden for legal aid and information. Many of the affected people portrayed unexpected decisions, mental illness, homelessness, violent deportations, inhumane treatment, and separation from family members. Despite knowledge of the harmful effects of these legal changes for individuals as well as for welfare professionals, the rules imposed had been defended by the Social Democrats, Sweden’s largest government party at the time. Some municipalities and civil society organizations had tried to protest, and there were calls for the government to initiate a critical review (it was demanded by, e.g., civil society, but never agreed to by the government). There was a significant need for collective action and legal mobilization.

Someone had to tell the truth to the people. Safe spaces were needed to enable silenced voices to come forward, and we needed to put an end to the extremely problematic legal developments in Sweden, from both a health perspective and a rule of law perspective. These ambitions, which came to lead the work of the Commission, would prove to be as productive as they were complicated.

The above passage describes the founding of the Asylum Commission, an unprecedented collective legal mobilization initiative that was active in Sweden between 2019 and 2022. It was established to investigate the harmful effects of legislative changes in asylum policies and the individual human right to asylum. Migration scholars, civil society activists, and welfare professionals were invited to take part. Such a diverse group – not only in terms of occupation but also age, educational background, and experience of asylum law and border controls – had the potential to both provide a deeper understanding of the effects of the new legislation and enable the circulation of relevant knowledge to the broader public.

An important issue with regard to my own role in the Commission, which I did not think much about at the beginning but which became increasingly important as time went on, and which also guides the present article, concerns expectations and the respondibility of me as a university professor in Sweden. This issue was linked to how we could provide a credible account of the consequences of Swedish migration law post 2015 that could be widely circulated and have an impact on legislative debates and changes, and at the same time take a clear stand in solidarity with those who had been left out of the legislative process and public debate, protection seekers themselves.

In this article, I approach this question through an analysis of two dilemmas encountered in the legal mobilization work. The term ‘dilemma’ denotes that there are no clear solutions. I understand dilemma as an unsolvable internal contradiction or logical flaw in the Commission’s work. I will *first* focus on the role of migration law and its manifestation of harmful mobility controls, and the risk, when working with this law, of reproducing various forms of injustice. *Second*, I will focus on the internal and external expectations of researchers in the context of the Commission’s work, which were marked by an assumed risk that our role would be undermined if we appeared ‘too activist’. The latter question, regarding engaged or activist scholars, has been a subject of increasing discussion in recent years (see for example Oredsson, 2023 who discusses this in a Swedish context). Further, studies on the role of scholars in the field of legal mobilization in Sweden and the Nordics are rare, as are studies on legal mobilization in the Nordics in general (see Molavi, 2024 for an interesting exception).

I write this article in a tradition of scholarship of hope. As many others, I am inspired by scholars as Gloria Anzaldúa, Angela Davis, bell hooks and Havel Vaclav. They all enthused research that does not settle for critiquing existing systems of power and oppression but also want to offer alternative visions and pathways toward social justice, equity, and hope. Through their writings and activism, they have helped generations of scholars to engage in scholarship that fosters meaningful social change. As committed scholars in Western Europe today, me and my colleagues’ endeavor is to develop oppositional knowledge where we link research, a vision for a better society, and pragmatic strategies for realizing progressive politics (Hill Collins, 2013). From a sociolegal perspective, sociologist Patricia Hill Collins’ practical strategies, which I will come back to in the theory section, are particularly relevant, given the position of legal sociology as a field situated between social science and law.

Following scholars such as Hill Collins and Kimberly Crenshaw, and in the Swedish context Paulina de los Reyes and Diana Mulinari, my memories of the experiences of working in the Commission, in what follows are put in dialogue with research that aims to “fruitfully articulate academic knowledge with a political vision” (De los Reyes and Mulinari, 2020, p. 185). Being part of a community of engaged scholars I strongly believe we have a role in bearing witness to social injustices but also to circulate knowledge that potentially can contribute to change, i.e., to practically counteract continued injustices. In the Commission, this form of engaged research was a prerequisite for being able to implement the project. We were, from my horizon, obliged to take an ethical responsibility including “to engage in social actions outside of academia in response to what [we] witness” (Hartley et al., 2013, p. 22). Further, it was important to avoid the problem of academization. Because, as the Brazilian liberation educator Paolo Freire has insightfully observed, relevant knowledge arises through discovery and rediscovery, through the restless and hopeful exploration that humans pursue in the world, with the world, and with each other (Freire, 2014, also see hooks, 2014). The exiled Palestinian writer Edward Said in his *Representations of the Intellectual* (the Reit Lectures, 1996) argues in a similar way for what I understand to be an anti-elitist perspective on the role of the intellectual. Said mainly argues that intellectuals shall address as wide an audience as possible in a kind of public educational spirit. Further, intellectuals should break down the stereotypes and simplistic categorizations that are such powerful constraints on human thought and communication (Said, 1996).

I start below by developing the methodological framework of the article. This is followed by an overview of the Commission’s background, including relevant prior research. I then analyze two inherent dilemmas in the Commission’s work, with reference to previous research and the article’s theoretical underpinnings. In a concluding discussion, I summarize my findings and highlight aspects left unexplored. A final reflection concludes the article, offering some suggestions for future work beyond the scope of the present contribution. Throughout the presentation, I have included quotes from my personal notes from the work with the Commission in order to visualize my own reflections. As I have re-read and remembered our work, a power shift in politics has taken place in Sweden. An even more anti-immigration government has taken office, with even more repression targeting people in need of refuge. This has obviously affected my memory and view on the Commission. In the article’s discussion, I try to reason about what has been the effect of this.

## 2 Method, data, and analytical process

This article is based on notes contained in a Word file titled “ventilation file,” which I wrote throughout my work with the Commission. These notes consist of several hundred pages spread over three documents titled “societal developments,” “changes in the right to asylum” and “diary – the commission.” The notes contain drafts of articles, debate pieces and chronicles, my thoughts on suggestions from various people about tasks they thought the Commission should take on, and summaries of books and articles. Moreover, the documentation includes my reflections on my own role as the research leader of the Commission, as well as relational issues, deliberations on the right time to take certain actions, difficulties maintaining work-life balance, and the importance of keeping a sense of humor. The notes also features some frustrated, incoherent writings about conversations that took place within the Commission’s Steering Group and Executive Committee and within the wider asylum rights community, of which I am a part.

The method used for this article is autoethnography (Méndez, 2014), meaning that I describe my experiences from the position of an insider in the Commission’s mobilization work, in dialogue with previous research. Importantly, although I was part of the Commission throughout the whole process, and it is my voice that is heard in this text, autoethnography in this case is not primarily about reflecting on my own feelings. As opposed to evocative autoethnography, where the researcher reflects on a particular topic for the readership to connect with their experiences and feelings (Méndez, 2014, p. 281), in the present text my aim is to focus on certain dilemmas documented in my (at times emotionally charged and at times critically distanced) ventilation file. Accordingly, I follow sociologist Anderson’s (2006) analytical autoethnography, where empirical data is used to gain insights into a broader set of social phenomena than those provided by the data themselves. In my case, this means that I draw on my personal documentation from the collective work of the Commission, and describe and analyze my personal experiences in order to understand key dilemmas in the mobilization work. My autoethnography is an observational reflection that may contribute to a discussion about collective legal mobilization work in the context of increasingly restrictive asylum regulations, including the role of sociolegal scholars.

My analysis has taken form through repeated slow readings of my field notes. This revisiting of the experience of working with the Commission, was initially about rest, recovery and retrospective reflection. I, together with the people in the steering group and executive committee, had worked intensively for a good cause without creating the political disruption that we wanted. I read in my notes from that time:

We are very tired, this collaboration was exhaustive.

During this time, we had not talked much about the dilemmas I will address in this article. As time has gone by and I have talked about the Commission’s work in seminars, student organizations, university courses and conferences – my memory from the work, and feelings in my re-reading of the ventilation file, has changed. Frustration fades as I reminisce, and a sense of being part of a collective struggle at a difficult time takes over. While my memory work from the beginning mainly was about ethical issues and a conglomeration of feelings varying between fatigue, disappointment, and faith that our attempts to bear witness will be left for posterity, two central dilemmas crystallized, around which this article revolves. These highlight important trade-offs which I did not consider at the inception of the Commission, but which later I see became central to our work and that I argue is relevant for future initiatives of legal mobilization in Scandinavia, where sociolegal and legal scholars are involved.

In the next section, I present the theoretical premises for this article, from a scholarship of hope perspective.

## 3 Theoretical points of departure

This article is about how to approach law in legal mobilization work and the role of sociolegal scholars in contributing to progressive politics by producing and circulating knowledge that can effect change.

### 3.1 Writing in a scholarship of hope tradition

One of two theoretical premises for the present article is that critical thinking also involves conversing around how we produce knowledge, how we identify as university teachers, and how we bear witness in society based on this knowledge. Questions of how knowledge circulates and how we can avoid the problem of academization are, in my view, central to critical theory. I find feminist and social activist bell hooks’ critique of the ways in which academic institutions can sometimes co-opt or dilute radical ideas, distancing them from the lived experiences of marginalized communities immensely in this context (hooks, 2014). I follow De los Reyes and Mulinari’s (2020) argument in reference to black feminist scholarships, that academization takes form through distancing academic knowledge production from everyday life. Specifically, De los Reyes and Mulinari highlight how academization means to forget the importance of creating spaces where the theoretical is “embodied in both the lives and struggles of those to whom we are accountable for our intellectual labor (and survival)” (De los Reyes and Mulinari, 2020, p. 192). As I interpret their reasoning, in order to develop critical theories about society and social relations, two central components are the ground-up co-production of knowledge that can be of benefit to justice movements and the practice of bearing witness as academics. We can therefore not draw a sharp line between how researchers produce knowledge, on the one hand, and what happens to our findings when they are disseminated in society, on the other.

Besides aiming to work within a scholarship of hope tradition as this has been developed in the Swedish context by De los Reyes and Mulinari, this article is also written in light of my own previous attempts to explore engaged sociolegal research that tries to deconstruct a deeply unjust system (see for example Lundberg, 2021; also see Fischer and Jørgensen, 2021; Lundberg and Söderman, *forthcoming*). Such scholarship takes inspiration from the critical legal studies movement’s Crenshaw (2013), Hill Collins (2013), and others. Both Hill Collins and Crenshaw depart from the claim that lived experience is a criterion of meaning. This has also become my deep belief, going from law-school (1992–1997) and then for years having encountered migrants who had been issued with deportation orders through various legal aid activities.

Hill Collins and Crenshaw argue that social justice challenges should be of central importance in academic work. For sociologists, such as Hill Collins, this is particularly apposite because sociology as a subject is situated between the “ostensibly pure spaces” (Hill Collins, 2013, p. 93) of social sciences and the humanities. Actively seeking out African American women’s perspectives and producing scholarship in which African American women recognize themselves, are important aspects of the kind of intellectual activism for which Hill Collins argues. In my work with the Commission, the promises made by the law to provide sanctuary are central, and similar to Hill Collins’ view of sociology, I consider that the subject of sociology of law is situated between social science and legal science. For a legal scholar such as Crenshaw, social justice issues are central because while the law promises to protect marginalized groups from oppression and discrimination, it proves incompatible to understand, name and recognize experiences that are linked to several discrimination grounds. One aspect of the problem to not depict valid experiences, which she refers to as “the single-axis framework” (Crenshaw, 2013, p. 139), makes it important for academics to join forces with social justice movements. Because it enables a deeper understanding of experiences that the law cannot capture. In the Commission, various actors with diverse experiences, and roles which often overlapped, collaborated on a number of activities in order to provide a “faithful account of a ‘real’ world” (Haraway, 1991, p. 187; see further below).

### 3.2 Writing critically about Swedish migration law

Drawing on the above reflections, my political position is that scholars have a responsibility to produce and circulate knowledge that contributes to efforts that contest violent forms of re-bordering. The methodological starting point for my work is that we can gain deeper knowledge about the effects of asylum law by trying to understand the real-life experiences of those whose lives are directly affected by this law. This is an important complement to other attempts to explain why legislation has certain effects (for Swedish migration regulations, see for example Vanessa Barker’s insightful analysis, Barker, 2017).

Ewick and Silbey (1991) argue that how ordinary people experience and understand law, “as they choose to invoke the law, to avoid it, or to resist it” (1991, p. 737) is an essential part of the life of law. Whereas some experience law as distant and removed from everyday life, others see it as a kind of game, “an arena of contest” (Ewick and Silbey, 1998, p. 131) where laws always contain loopholes in which action can be taken to be able to reach a certain outcome. In this view, the rules are perceived as possible not only to be played *by*, but also to be played *with*. In that context it is important to understand the capacity through which law and the legal system can be effectively utilized to reach certain goals (Ewick and Silbey, 1998, p. 28, 151). Ewick and Silbey (1998) furthermore describe that taking an against-the-law approach leads to the mobilization of effective resistance to escape the unwanted effects of (legal) power.

To understand the life of law from the position of people seeking refuge in Sweden, something needs to be said about Swedish migration law as seen by the state. This legal area decides what right foreign nationals have, to stay in Sweden. The Aliens Act is based on the 1951 Refugee Convention as well as a number of EU regulations. Each asylum application is individually assessed based on this Act, by the Migration Agency. This authority first examines whether the applicant needs protection due to risk of individual persecution in their former home country. Second, it is assessed whether the person is a refugee because of an ongoing armed conflict, and third – in exceptional cases – whether there are distressing humanitarian grounds for a residence permit. The Migration Agency’s decisions can be appealed to a migration court and to the Migration Court of Appeal if there are special reasons. The whole process is based on the principles of rationality and neutrality which should be safeguarded by law and legal principles. In practice, as I will explain in the next section, the process is highly arbitrary and leads to much harm.

## 4 The political context and previous research

### 4.1 Activist migration research

As I write this article, in the winter of 2023, Swedish migration regulations have recently gone through a paradigm shift which scholars in the Asylum Commission have investigated and tried to contest in collaboration with or alongside the migrant rights movement (Lundberg et al., 2021; Elsrud et al., 2021a,b). Similar activities have also emerged in other parts of the world, through initiatives such as Academics for Refugees (2023), *the Silent University* in various European cities (The Silent University, 2023), *Freedom University* in the US (Freedom University, 2023) and the Turkish *Academics for Peace* (Korkman, 2022). Initiatives such as the above are paralleled by a heightened acknowledgment of the necessity for university educators to take active responsibility in supporting social and political transformation (Torres, 2019). Especially in the field of migration regulations, there are many reasons why academics need to stand up for a solidaric migration policy. We can straightforwardly observe mounting violations of the right to asylum at the EU’s external borders through (silent) acceptance of drownings in the Mediterranean, pushbacks (Casas-Cortes et al., 2015), the harmful effects of the so-called EU-Turkey deal (Heck and Hess, 2017), as well as proposals to move states’ asylum processes to other countries (Limb, 2022) and calls for states to withdraw from the Refugee Convention (Philo et al., 2013).

Simultaneously, there is also a lamentable acknowledgement amongst critical scholars that asylum in its institutionalized form is a deeply racialized construction (Achiume, 2022). The right to asylum, as negotiated in international agreements such as the 1951 Refugee Convention, on which EU and Swedish law is based, is a system where migrants are perceived as they have fled for the wrong reason and are too many (Achiume, 2022). Systems of mobility controls such as the Swedish asylum process, are, at their very core, based on an assumption that migrants are threatening to the security of people in high-income countries (Achiume, 2022). Therefore, central problematic dichotomies in debates around asylum, such as refugees versus economic migrants, will not be resolved by, for example, expanding the refugee category to include more protection needs (e.g., due to poverty). As Tendayi Achiume has pointed out, such measures are incapable of addressing the heart of the problem: that individuals seeking a new home are perceived as outsiders with no legitimate claims to equal rights (Achiume, 2022). This problem, which relates to the uneven global distribution of the right to migrate, has also been brought to the forefront in research on the Swedish asylum process, particularly in discussions about the blurred line between economic hardship and the widespread denial of economic, social, and cultural rights as a motivation for seeking refuge as opposed to political oppression as the reason for forced migration (see Joormann, 2019).

### 4.2 Sociolegal activist scholarship

There are signs that researchers in general are becoming increasingly engaged and taking a stand for social movements, climate researchers being a case in point. Sociologist Fernando Tormos-Aponte and colleagues recently concluded from a literature review and a survey study that scientists all over the world are increasingly speaking out on various political and social issues related to their own research fields and in solidarity with social movements (Tormos-Aponte et al., 2020; for examples of “activist research” in Sweden, see Lind, 2020, ch 3 and Söderman, 2019).

Looking at sociolegal research in particular, while there have been studies on the interplay between law and social change (see McCann, 2006; Lehoucq and Taylor, 2020; Sager and Kolankiewicz, 2022) and collaborations between social movements and legal professionals (such as lawyers, see Sarat and Scheingold, 2006), there has been little interest in activist research as such. Sociolegal scholars generally do not explore their own participatory role in the ways that feminist researchers and anthropologists have done (Speed, 2006; Huschke, 2015; Gutierrez and Lipman, 2016; Loperena, 2016; Mattos and Xavier, 2016), i.e., to partake in knowledge production from situated positions (Haraway, 1988). In this respect, the present article is a contribution to sociolegal research on legal mobilization.

### 4.3 Activist sociolegal research in the Nordics

Regarding the Swedish context, it is important to understand that a collaboration such as the Asylum Commission, where academics strategically and openly collaborated with actors outside of academia to produce critical knowledge, and then tried to circulate this knowledge in society, is rare (for an exception besides the Asylum Commission, see Westlund et al., 2016). During the egalitarian (Therborn et al., 1978) and internationalist (Karlsson Schaffer, 2020) era of the 1960s and 70s, academics in Sweden were more active in progressive political work than today. At that time, one example of such engaged scholarship took the form of a sociolegal conversation around what function law should have in the development of progressive politics. An important starting point for this debate was an intervention by the sociolegal scholar Eriksson (1980) who from a Marxist perspective advocated for legal scholars to reflect on the function of law in a society divided into classes. In such a society, law would be guided by certain specific (social and political) objectives; namely, to fulfil the social and political needs of its members. The debate sparked by Eriksson’s article was intense and took several different directions (see Hydén, 1982). On the one hand, a legal optimism movement took shape which aimed, as Eriksson had suggested, to take law “at face value” – i.e., to take its promises regarding labor, equality and social legislation seriously (Gustafsson and Vinthagen, 2011). On the other hand, Thomas Mathiesen, among others, warned against taking legal argumentation into political conflicts. The danger, argued Mathiesen, is that lawyers let the law take the upper hand and thereby stifle political conflicts by dressing them up in legal terms. As law, by definition, is repressive, legal mobilization actors should avoid working with it and instead consistently ignore it (Mathiesen, 1982; also see McCann, 2017). Sociolegal professor Håkan Hydén criticized Mathiesen and argued for a nuanced understanding of the relationship between law and society, because, as he stated, law could *potentially* change society for the better, and law could be utilized as an important instrument for change. By understanding, investigating and/or using the law as a tool for action, which was what Hydén believed law was fundamentally about, Mathiesen’s dogmatic view on law and Erikson’s doctrinaire loyalty to legal arguments lost importance. Hydén argued that the important thing was to:

[…] encourage and persuade individuals in society to make demands and fight for their material rights independently of the legal superstructure [so that] human needs can be set against the demands of the system in the pure form that the conflict appears to the individuals involved (Hydén, 1982, p. 34–35 my translation).

Over time, these debates petered out, and academics in the Nordics became less focused on taking normative stands or working openly for social change. There are several reasons for this development, one of which concerns changed conditions for research. In light of increased administrative duties and insecure employment conditions, combined with greater competition for external research funding and higher production requirements in terms of peer-reviewed publications, public engagement has lost ground in relation to traditional social science research (Gruber and Lundberg, 2021). Similar developments have been seen within feminist critical studies in the Nordic context. Svensson et al. (2004) discussed in the beginning of the 2000s how the feminist legal tradition in Sweden had distanced itself from the women’s movement, becoming more concerned with internal relations between feminism and jurisprudence (Svensson et al., 2004). In the early 2020s, despite longstanding tensions between activist research and academic success (Cancian, 1993), things seem to be changing. This, I believe, undoubtedly has to do with the broader political development. Given the current legislated proposals, engaged legal sociologists and sociolegal scholars are essential actors in the ongoing debates. I reflected on this in my diary notes in the following way:

*Why do we who are active within academia never discuss the cost of not engaging when humanistic values are at stake? Why is the activist label so often used as a tool of power to silence? This happens also within the Commission, where we are all counted as activists? Why do I so often doubt what we are doing? It is difficult not to succumb to self-censorship.*

In the next section I present the back ground to the Commission.

## 5 Why an asylum commission?

### 5.1 The background

In early 2019, I was approached by a prominent activist in the refugee rights movement in Sweden to initiate a collective trans-sectional critical review of the legal development in Swedish asylum law. I was then a newly appointed professor, with a tenured position at a progressive university in Sweden. My new workplace was characterized by a welcoming atmosphere and multidisciplinary research regarding social issues. In conversations with colleagues who were, as myself, concerned about the current situation in Sweden (many of whom were also engaged in the migrant rights movement), I saw an opening for developing new platforms where collaborative critical knowledge production could take place. We could conduct research together with actors outside of academia, contesting the increasingly harsh migration policy since 2015, was timely.

As I illustrated in the introduction, when we initiated the Commission in 2019, it was a time of upheaval in Sweden. Since late 2015, asylum law and refugee reception had gone through several changes following the government’s U-turn on asylum policy in November 2015. During 2014–15, the number of refugees seeking protection in Sweden and Europe had increased due to escalating armed conflicts in 2014 and 2015, particularly in Syria and Afghanistan. A solidarity movement had sprung up all over Europe, perhaps most clearly manifested in the slogan “Refugees welcome” (Povrzanović Frykman and Mäkelä, 2019).

Attitudes towards refugee reception however changed when the Swedish government made its turnaround near the end of 2015. Government representatives spoke daily of an impending systemic collapse. In early 2016, transport companies were banned from taking passengers without ID documents to Sweden (Swedish Radio, 2015) and a temporary law was quickly prepared with the explicit aim to greatly reduce the number of asylum seekers (Act 2016:752). The Temporary Asylum Act was adopted by parliament in June 2016, making temporary residence permits the main principle for everyone seeking protection, and humanitarian reasons as grounds for asylum were withdrawn except for exceptional cases. (I will return to this temporary law, as it was important for the Commission’s work.)

Other legislative changes introduced were restricted financial support and obstacles to family reunification through increased dependency requirements and a narrower definition of who constitutes a family member. Further, in the spring of 2016, a readmission agreement was concluded between the Swedish and Afghan governments, opening the way for more deportations to that country which had for several years been the most dangerous for children to live in worldwide (Hertzberg, 2021). Furthermore, the government gave the Migration Agency a mandate to “re-age” young unaccompanied minors more extensively and at the beginning of the asylum process, which led to thousands of young people becoming formally recognized as adults and thereby easier to deport (Scott, 2022). The methods used for these age assessments have been criticized by legal scholars as “junk science” (Noll, 2016).

Not all legislative processes were moving in the same direction, but proposals taking a more pro-refugee direction never reached the stage of actual voting in parliament. One example was a state enquiry that I was involved with as an independent investigator about impediments to enforcement of deportation decisions, which was presented in 2017 (SOU, 2017:84), demonstrating the shortcomings in the current regulatory framework and legal practice. However, the proposals from the inquiry, which included the granting of residence permits in case of practical barriers to enforcement in more cases, came to nothing. The same fate befell another government inquiry on establishing legal routes to Europe for people in need of protection (SOU, 2017:103).

### 5.2 High ambitions in a time of limited opportunity structures

In light of the range of measures implemented to make it more difficult for asylum seekers who reside in Sweden, and signal to potential new groups of asylum seekers that they were not welcome, the Asylum Commission was operating at the worst time possible, in terms of having its demands and claims heard. Legislation and the political debate were going in the wrong direction, but nevertheless, or perhaps precisely because of this, the gathering of testimonies and the strategic mobilization work we conducted were more important than ever.

The Commission’s goal, based on the theoretical premises presented above, was to conduct a review of legislation and law enforcement affecting people who had sought asylum in Sweden between 2015 and 17, drawing on real-life testimonies in order to co-produce critical knowledge with a political vision. Thus, we were working with an egalitarian approach. This explicit ambition was an attempt to decenter our activities away from national asylum regulations in a way that deemphasized the state itself (McCann, 1998, p. 248) and “to shift the ownership of knowledge from the researcher back to those who experience the research phenomena” (Bilotta, 2020). Further, our goal was to challenge the “oppressive structures and power asymmetries that underpin migration contexts,” including research on migration (Clark-Kazak, 2021, p. 131). In being guided by those who were “living the law,” and decoupling law from the state, we aimed to unpack preconceptions about Swedish bureaucracy as legally certain (*rättssäker* in Swedish, a concept that encompasses both predictability and an ethically reasonable outcome). From my perspective, we were a collective of sociolegal knowledge seekers and knowledge producers.

### 5.3 Activities in the asylum commission

The explicit goal of the Commission’s activities was to critically review previous and ongoing developments in the field of Swedish asylum regulations, such as the right to residency and access to welfare rights and participation in society, and to influence legal developments going forward.

The Commission involved researchers, civil society representatives and professionals. Over 3 years, we collected testimonies from asylum seekers, including from inside detention centers (Lindberg et al., 2022); interviewed nearly 100 people (Asylum Commission, 2022b); conducted in-depth analyses of asylum case files (Lundberg et al., 2022); put together a timeline mapping the development of restrictive legal measures (Asylum Commission, 2022c); and published a shadow directive to the Swedish government (Asylum Commission, 2020) and an anthology including 24 chapters written by asylum seekers, researchers, and activists, several of whom also had personal experience of seeking asylum in Sweden (Elsrud et al., 2021a,b). A direct channel for the circulation of testimonies was established in the form of a blog that allowed people to share their experiences in their own words (Asylum Commission, 2023).

A key part of our legal mobilization was the temporary law that had been adopted in June 2016. The Temporary Asylum Act had fundamentally changed what it meant to seek protection in Sweden (Lindberg, 2023). When the decision to extend the law was up for debate (e.g., in early 2019), civil society organizations expressed strong criticism on a number of points. Among other things, they declared that the law was in sharp contrast to the European Convention on Human Rights and the UN Convention on the Rights of the Child (Rädda barnen, 2019); that too much responsibility for how the law should be interpreted had been transferred from the legislator to the practitioner, resulting in arbitrariness and uncertainty (Rådgivningsbyrån för asylsökande och flyktingar, 2019); seriously ill children who would have been granted permanent residence permits under the ordinary Aliens Act were no longer allowed to stay; and there were increasing problems with discrimination against vulnerable groups as a result of increased requirements for self-sufficiency (FARR, 2019).

In this context, the Commission wanted to explore questions such as: What is it like to encounter Swedish migration law in practice? What problems with the temporary law do asylum seekers themselves experience and how could these experiences be made visible in the public debate? What harms do the current changes in migration law cause, for people who seek refuge and for social relations more broadly?

The Commission ended in August 2022 with an international conference where activists and researchers from various border areas around Europe came together to listen to each other’s experiences and through a collective writing exercise, imagine a different future (Asylum Commission, 2022a). As part of the writing exercise during the conference, we formulated a manifesto of a different migration politics that was filmed in 17 different languages (Asylum Commission, 2022d).

On my part, I had for many years been thinking about the issue of how scholars can work in solidarity with people who are directly targeted by legislative changes and whose experiences of asylum regulations are ignored, and how our collective work to both invoke and resist the law might bring about political and legal change. I had also tried to work in this direction, sometimes with colleagues around me questioning the approach. In my analyse for this article, I have returned to these questions by conducting an analysis of two key dilemmas encountered in the Commission’s work, which I present in the next section.

*I have to remember my recurring experience, and that this has been the case throughout my academic life, that the most profound analyses, the most relevant insights into what migration law as a legal field entail when it operates, came from encounters with those affected by its operation: undocumented migrants, asylum seekers, people facing deportation.*

## 6 Analysis

### 6.1 Working within legal arenas, with law and beyond the law

When I began working in the Commission, it became clear to me what McCann has observed in other contexts: that civil society activists tend to “embrace the basic ideals of the legal system as desirable goals leading their own political activities” (McCann, 1991, p. 238). More specifically, in the case of the Commission, the right to asylum was perceived as an ideological political resource in that it promotes ideas about how things ought to be done (see Scheingold, 1974). The legislated right to asylum was therefore naturally an important value in the Commission. However, while we were prepared to fight for this right tooth and nail, as a critical scholar I was also aware of the problems linked to this ideal, outlined above with reference to Achiume (2022).

A related problem – that that I also reflect on in my diary notes – was the problem, that the wider migrant rights movement is more likely to achieve its goals if it stands “free from the constraints imposed by law and lawyers—even the politically astute ones” (Brown-Nagin, 2005, p. 1502). As things would unfold, lawyers involved in the Commission did not pose any difficulty in this context. At an early stage, those lawyers who had expressed an interest in the Commission decided that they could only participate as (independent) members of a reference group, considering the openly critical perspective that characterized the Commission’s work. Active participation as a commissioner would include taking a critical stand in the public debate, and this might affect the lawyers’ clients in a negative way. Instead of being directly active, the lawyers became members of an external reference group that could provide testimonies about their experiences that were valuable for our activities.

While expertise in terms of professional lawyers did not constrain our work, the law and its promises did. More specifically, the dilemma we faced was linked to our goal of stopping the temporary law from becoming permanent. This, as it were, inevitably meant that we argued for the ordinary Aliens Act to be reinstated. The Aliens Act – adopted in 2005 – in contrast with the content of the temporary Act even though this law also had shown to have harmful effects (see for example Wikström and Johansson, 2013).

Many of the commissioners, including myself, had previously conducted research on the limitations of the Aliens Act and had seen closeup its violent consequences in our volunteer legal aid activities for asylum seekers (Lundberg and Söderman, 2024). These experiences had taught us that migration authorities and courts applying the ordinary Act are not, as a general rule, guided by the right to asylum, but rather by a quest to find reasons for issuing deportation orders. This tendency, to actively find reasons for a rejection has been described in depth in previous research and is often referred to as a manifestation of a “culture of disbelief” (see for example Lundberg, 2020; Atak and Crépeau, 2022).

With the introduction of the temporary act, the miscrediting of people seeking refuge increased, which was likely an effect of an increasingly intolerant media discourse that was palpable at the time (see Ekman and Krzyżanowski, 2021). In the first instance, the temporary law had limited the room to grant residency. Secondly, it enabled the authorities and the courts applying the law to interpret social facts and regulations in an increasingly restrictive way. These developments in Sweden have been described in comparative studies as:

… inward and outward measures of deterrence, such as high rejection rates, subsidiary protection regimes, the introduction of border controls and the increasing pressure of return (Garvik and Valenta, 2021, p. 13).

The culture of disbelief may also be understood as a form of organizational logic sprung from the deeply racialized border regimes that Achiume points to in her work.

Against this backdrop, an obvious dilemma for me concerned the exclusionary mechanisms regarding who would be subject to the protection by the law should we succeed in stopping the temporary law. *This bothers me deeply*, I wrote in my diary. The commissioners, including myself, as well as the research participants (interviewees and people sending spontaneous testimonies), often fell into certain ways of talking.

*Frequently, we talk about asylum in a way that reproduce a circumscribed definition of who has earned protection. It is the persecuted, vulnerable individual, the one who is most in need of humanitarian assistance; or those who have earned the right to live in Sweden by establishing themselves in the labor market while simultaneously learning Swedish; or those who because of their age are part of a group that is needed in this country for economic reasons (i.e., due to the ageing Swedish population requiring care).*

These categorizations were often not as explicit as I described in my documentation, but they seeped out as underlying arguments when we discussed, for example, that such and such a person must now be allowed to stay, or that such and such a group absolutely could not, reasonably, be deported. Retrospectively, now that time has gone by, I realize we were caught in a language of racial borders, and in the words of Achiume, the core purpose of such borders is to enforce exclusion or inclusion on a racial basis.

Achiume invites us to think about race itself as a special kind of border. In the contemporary migration context, it is used as a border control device to police people’s belonging to certain spaces and territories. Non-White people are targeted in a spirit of a “racialized presumption of illegality and outsider status” (Achiume, 2022, p. 486). Whiteness, by contrast, “functions as a mechanism of presumptive inclusion … and mobility facilitation” (Achiume, 2022, p. 486). These much relevant reflections were difficult to consider in the Commission’s legal mobilization, for several reasons. Nor did we take much time to reflect on the obvious risk of reproducing a racist system as we mobilized against the temporary law. Inconsiderately, we allowed to a large extent for this law to set the (limited) framework for our work. In retrospect, I have realized that we did not problematize the law enough, although I posed questions about it in my notes:

*Should we ignore the law altogether and work with other mobilization strategies* (see more on this in Mathiesen, 1982 and McCann, 2017)?

In fact, we could not disregard the law, for very specific reasons.

Despite the anti-immigration drift and the shortcomings of the legislation, our ambition to stop the temporary law did not mean that we could ignore it. On the contrary, we were forced to work “with the law” (Ewick and Silbey, 1998). The main reason for this was that people who approached the Commission themselves, or whom we contacted to ask if they wanted to participate in an interview, were in urgent need of help. Providing legal aid early on became a standard part of the Commission’s interviews. Besides individual legal aid, we started to offer group sessions where specific problems were discussed, such as practical impediments to enforcement and the room in the law to grant residency due to such barriers (these sessions ended with the pandemic). Being stuck in the position of both trying to make use of the temporary law (when assisting asylum seekers), and at the same time trying to stop it based on lived experience of it, amounted to two completely different strategies.

Remembering the discussions in the Commission and re-reading my notes, I think I understand that working simultaneously with and against the law was a necessary political and ethical response to the situation in Sweden at the time and considering the constitution in the Commission. Not least, this double approach was a matter of building trust between researchers and people testifying about their experiences, and a redistribution of legal knowledge. I also see, in hindsight, that working with the law enabled a more in-depth understanding of its shortcomings. I had known this before from my experience with legal aid in closed rooms. This time, the work on the law was happening more openly, not in safe spaces without the scrutiny of the establishment pushing for more repressive policies.

*Our attempts to provide legal aid sessions seem to be very productive in that they help us understanding why the law needs to be stopped. In this respect, the sessions are one prerequisite for identifying relevant problem areas that then need to be analyzed in detail in the next step. In addition, the legal sessions are a way of giving back directly to those in need of help, and to somehow recognize before them, that we see these injustices. To show that we listen to you.*

Similar reasonings, but in a context of broader antiracist activism in Sweden, has been explored by feminist researchers Maja Sager and Marta Kolankiewicz (Sager and Kolankiewicz, 2022). They show that the law, in the context of antiracist struggles, presents opportunities to re-politicize the legal arena by highlighting that law is neither neutral nor objective. The law may also empower, through the redistribution of knowledge about formal rights and how to claim those rights. These ways of using the law, Sager and Kolankiewicz explain, emerge from an ambivalence towards “the ideological commitment in the critique of law and their position from which it is impossible to ignore the legal arena” (Kolankiewicz, 2022, p. 534).

Besides legal aid sessions, for the Commission the legal arena was also constituted by an ongoing parliamentary inquiry into whether the temporary law should be prolonged and which parts should become permanent. We decided to approach the legislature through a collective writing and lobbying exercise, aspiring to foreground other perspectives than those that were currently dominant (see McCann, 1991, p. 244). The exercise took the form of rewriting the government’s directive to the parliamentary inquiry. The official state inquiry included representatives of all political parties in the Swedish parliament, and it had been assigned by the government to propose an Aliens Act that was very similar in content to the temporary law (see SOU, 2020:54). The Commission collectively rewrote these directives, as if we were the government posing questions to the inquiry which from our perspective *should* have been posed (theatrics being well visible in our text). For instance, given how unaccompanied children had been harmed by the temporary act and its legal application, we commissioned the inquiry to suggest restorative treatment for these individuals. Given the increasing numbers of people residing in Sweden with no right to remain while also having no possibility to return, we instructed the inquiry to come up with proposals so that *more* people would be granted residence permits if they were stuck in limbo (similar to the above-mentioned inquiry 2017:84). Our directive also included hundreds of references to scientific studies to justify the assignments and promote a research-based transformation of the legislative process.

The shadow directive is one example of the Commission’s work in the legal arena which at the same time goes beyond the dominant understanding of who should be protected within this arena. From my notes:

*From my point of view, this collective writing exercise is one answer to the question of how to mobilize as a collective in a way that disrupts the abusive, repressive, exclusionary, and dehumanizing migration system that is constructing and controlling deportable populations. This work can help us to think beyond the current violent system (read Achiume on this).*

Many of my notes are about when in time it was appropriate to do a particular activity but there are also reflections that are about motivation, reminders of what we did and why:

*The Shadow Directive should culminate in a task to develop proposals for the progressive politicians to use as they argue. We should be ready by mid-June, well before the proposal from the Migration Inquiry to be presented in August. How should we reach out? No Järva Week and no Almedal Week this year? [because of the pandemic] Can we have a completed draft ready for the steering group to review by May 12^th^? We did announce that a document is in the process of being written and that the commissioners will provide insightful feedback. They can be reminded again in May …*

Another way of thinking about the Shadow Directive, which has become clearer to me as I have re-read to my field notes and drafts, and remembered how many we were in this process – many people provided feedback, references, and suggestions for improvements – is that what we actually did was to formulate a kind of micro-utopia. Through the collective writing process we put down in words what might be possible had we lived in a different time. In other words, we took a utopian approach grounded in the present, to imagine the future as we wanted to see it, knowing full well that this was not possible in the time we now live in.

In conclusion, the Commission’s work with the law to support individuals and groups, and our work in the legal arena, did not allow us to formulate, or even reflect much upon, the need for a radical critique of a regulated border control system. To a limited extent – by writing the Shadow Directive (and formulating a manifesto at the Commission’s final conference; see Asylum Commission, 2022a) – we managed to reach beyond this system. However, I cannot escape the fact that our mobilization activities ended up confirming McCann’s observation. I quoted him in my notes:

*“legal relations … tend to be double-edged, at once upholding the larger infrastructure of the status quo while providing limited opportunities for episodic challenges and transformations in that ruling order”* (McCann, 2006, p. 19).

### 6.2 The role of academics and academic institutions

My second dilemma revolves around my own position and the role of academic institutions in the Commission. As mentioned in the introduction, I had recently taken up a position at a progressive university in Sweden and I found myself in a group of colleagues who wanted to work in co-operation with the surrounding community. Still, considering the Swedish view on academia, it was challenge for me personally to co-produce knowledge:

*My wish, in the spirit of Hill Collins, is to develop oppositional knowledge, linking research, a vision for a better society, and pragmatic strategies for realizing progressive politics* (*see more on this in* Hill Collins, 2013). Why is this so difficult in Sweden?

From a sociolegal perspective, the pragmatic strategies that Hill Collins describes and which I noted in the theory section of this article, were particularly relevant, given the position of legal sociology as a field situated between social science and law. These reasonings are mirrored to some extent in my above description of how we worked with migration law.

The practical work and mobilization strategies also involved a great deal of thinking on my own position in relation to the expectations that emerged regarding this role, both internally in the Commission and externally in relation to other actors in society.

Scholars and others who have inspired my academic work in general, not least within the scholarship of hope tradition, argue that it is a political and moral responsibility for academics to develop knowledge with a “political vision” (De los Reyes and Mulinari, 2020, p. 185). As for the researchers involved in the Commission, none of us were grounded in a strict positivist ideal of science. As critical scholars we shared the stance that science is always political. Nonetheless, we had to consider the expectations of academic work in society, and a dominant positivist scientific ideal which also permeated the public debate around migration law, where knowledge obtained through quantitative information, observation and systematic processing as a rule is considered more reliable and therefore more effective than partial knowledge (Haraway, 1988).

*It is difficult when we are expected to provide facts drawn from a distanced positivistic position. Considering these expectations: I wonder, how should we make a change?*

In a way that seems naïve in retrospect, I imagined that the different groups within the Commission would work closely together, admittedly with different roles and backgrounds. In our differences, we would inspire each other with humility and respect, I remember I was thinking. Many of those involved in the Commission also had common experiences of being both activists and researchers, and many had a migrant background. From my position it was fair to call us all activists because activism, in the sense that we wanted to bring about political and social change, was a common denominator and an important reason for those who joined the Commission to do so. Simultaneously, the Commission comprised a spectrum of stakeholders, encompassing not only academically inclined researchers doubling as activists, but also civil society advocates, exhibiting varying degrees of radicalism in their perspectives on migration law. This dynamic presented novel challenges. I believed that aligning multiple, diverse actors and interests would make us stronger and more capable of effecting change and thinking beyond the current system. Through a multiplicity of perspectives and true popular intellectual work (Said, 1996), we would be able to reach the broader public. The ethical and moral responsibility to act also needed to be coupled with reflection on the impacts of the actions taken. I frequently expressed my abundant doubts with regards to these impacts in my diary entries:

*How can we as a diverse collective provide a truthful account of the consequences of post-2015 migration law that will have a bearing on legislative debates and changes, while reconciling conflicting perspectives and expectations among the actors involved, without compromising the ambition to take a stand in solidarity with people seeking protection in Sweden?*

This question of how to bring about change in society has been discussed by a number of researchers. Migration scholars Leandros Fischer and Martin Bak Jørgensen, asking what we try to do when we do migration research, identify the problem of ethical discussions that focus only on respecting individual autonomy and ensuring equitable sharing of benefits. Such requirements, Fischer and Bak Jørgensen argue, are not adequate for describing action-research approaches aiming to intervene and spur social change. Drawing on Maurice Stierl, they instead call for taking an engaged and critical research position that, at least in our imagination, reaches beyond vague principles such as “do no harm” and instead focuses on how to deconstruct a deeply unjust system (Fischer and Jørgensen, 2021, p. 151).

For Haraway, the ambition to contribute to progressive politics involves the attempt to produce a “faithful account of a ‘real’ world” (Haraway, 1988, p. 579). The Commission made claims to tell the truth in this Harawaysian sense. More specifically, we wanted to reveal the truth about the material consequences of the temporary act. Such a truth could be revealed, I assumed, and it could be accessed by talking and acting with and on behalf of asylum seekers who had been directly targeted by the law, what Haraway frames as “the privilege of partial perspective,” i.e., the position of being in the asylum process (Haraway, 1988).

This approach required us to be as radical as possible and as cautious as necessary, at the same time. We would be uncompromising in revealing the actual consequences of the legal changes through as many channels as possible, but careful to do so sensibly so that people and policymakers would listen to our truth telling. In this balancing act, linking up with the asylum rights movement’s broader resistance and continuous advocacy work was an important step.

The project started off well in the sense of engagement and expectations of what the Commission would be able to achieve. Following the initial meeting of the Commission and the publication of our opinion piece (see the introduction to this article), we received some 60 spontaneous testimonies by letter, phone or email, including longer case descriptions of problems in the Swedish migration administration. Each person who wrote to the Commission’s university email address received an automatic reply thanking the person for contacting us, that they should not send us any documents (i.e., from their asylum case files) and that we would get back to them in due course. It was also stated that we were not able to offer legal aid on individual cases. As explained in the previous section, the latter was impossible to uphold legal aid was absolutely necessary.

The dilemma concerning the role of researchers crept up in meetings between the Commission’s various groups concerning how the testimonies should be systematized and circulated. An internal debate arose around how we should fulfill expectations of so-called neutrality and at the same time ally ourselves with asylum seekers by taking a clear stand against direct verbal attacks in the public debate and degrading treatment by the asylum authorities. Actors, such as elected officials and major media outlets that reached a large part of the population were indeed more interested in objective measurable facts or stories from those who could be described as either victims or self-made champions.

In my diary notes after one of the meetings, I listed what people had said:

*we must not be too judgmental, value-laden words should be deleted, we must be careful not to get too ideological*.

Plainly, just as the credibility of asylum seekers is often questioned in the asylum bureaucracy, several people in the Commission assumed that our work would also be questioned if we did not stick to using neutral language. I felt that those among us who were researchers within university settings suddenly were getting an important role in ensuring that the Commission remained trustworthy as in neutral. We represented, I remember re-reading my notes, a kind of proof of validity in terms of not only methodology and theory, but also in terms of telling the truth to power. The expectations of several civil society actors regarding the role and actions of a researcher clashed – I see hindsight – with my epistemic standpoint and my perspective on how I wished to engage in public debates.

I need to also remember the societal debate. The politicians drafting the proposed legislative changes at the time of the Commission’s work kept rushing to develop new, more restrictive legislations. They did not consider previous research on what constitutes a solidaric asylum reception. The focus of the debate was predominantly on how to keep refugees away. While taking a stand as engaged scholars, against this backdrop, was seen as risky because it could mean that we did not get the impact we wanted, it was also necessary to explicitly align ourselves with asylum seekers as they were completely excluded from the debate. Their experiences were ignored, their voices silenced, and as non-citizens they did not have legitimate ground to hold politicians accountable.

*I do not want to contribute to further silencing those directly affected although I feel this is what happens. I want to make visible the experiences we have had shared with us. It would be a mistake to let the means justify the end now by taking a positivistic position.*

My personal documentation here testifies to the difficulty of taking an explicit position as a ‘truth teller’, in terms of disseminating knowledge in a ground-up alliance with people who had sought refuge, while simultaneously maintaining an appearance of the expected neutrality. This dilemma could not be solved, but neither could it be ignored. As Hill Collins points to, it is difficult to emphasize these different sides as a committed researcher. My experience is that they these sides are also extremely difficult to uphold in practice, especially in a highly politicized field such as migration law (see also Clark-Kazak, 2021).

*If we do not manage to handle this tension wisely, we would only serve to further reproduce the silencing of the voices of people seeking protection.*

## 7 Discussion

In the present article, I aim to make an empirical contribution to scholarly and public discussions about collective legal mobilization in contestation of violent forms of re-bordering in Nordic welfare states post 2015. I also want to reflect on what role critical sociolegal research may play in this context by reflecting on dilemmas relating to ethically and politically responsible sociolegal scholarship. In a broader perspective, the article addresses the question of how higher educational institutions and researchers can, or ought to, act in politicized fields such as irregular migration. To fulfil my aims, I have investigated my own field notes about the work of the Asylum Commission and focused on two embedded internal contradictions, so-called dilemmas.

*First*, I have discussed how the Commission had to maneuver the possibilities and shortcomings of Swedish migration law, more specifically the possibility to stop the temporary law from becoming permanent, thereby unavoidably reproducing an image of the “ordinary immigration law” as something desirable, even though we were fully aware that it was in essence a (violent) expression of border controls. Our strategy was to work through the law beyond the law. Law, as individual legal application, might not be a valid tool for structural change. However, legal arenas could provide a space for structural change where one can also reach beyond the limitations of law. At the end of the day, law is an important institution in society for dealing with various common concerns. As I noted in my diary:

*from the perspective of people seeking protection in Sweden, to ignore the law is not an option. Asylum seekers have no choice but to work with the law at least in their own individual case.*

Thus, despite prior knowledge of legal application as harmful, we had to grapple *with* the law; this was an unfortunate necessity for asylum seekers and therefore also for us as engaged researchers (Sager and Kolankiewicz, 2022).

The challenge was to find out how we could both work with the law and use the legal arena and the political opportunity structures to reach beyond the law’s exclusionary elements. The *Shadow Directive* and *The Manifesto* here became a means to shape the future we envisioned, grounded in the current situation. My analysis raises questions about the risk of inadvertently reproducing exclusionary effects in legal mobilization. To cite Wendy Brown (who also appears in my diaries in several paragraphs), mobilizing for the right to asylum with reference to promises made in national law, at best may function as “an indisputable force of emancipation at one moment in history” and at worst become “a regulatory discourse—a means of obstructing or co-opting more radical demands or simply the most hollow of empty promises” (Brown, 1995, cited in McCann and Lovell, 2020, p. 368–69).

*Second*, I have explored the question of who is esteemed as a knower of law and which forms of knowledge is legitimate in society, as anticipated in my role as a sociolegal researcher (and a figure in the Commission). As engaged scholars, there is always a tangible risk that we will reinforce what we want to contest. One aspect of this risk concerns how what we say and do is perceived in society, and in our case by politicians and the legislature.

Today, when I go back to my notes in the ventilation file, I see more clearly that the co-creation of knowledge required the dual role of activist and researcher. While the visibility of this role can always be hijacked by repressive forces to try to undermine the contribution of critical researchers. This is a problem that, as I revise this text in the light of comments from reviewers, I see before me with all due clarity. The freedom of academia is under threat, in Sweden and in other countries (Scholarships of hope, 2023). At the same time, repression is more prominent than during the time when the Commission’s activities took place. Therefore, engaged research is even more important and more difficult.

In any future trans-sectional legal mobilization initiative that seeks to form broad alliances in contestation of the harms of migration law, I wish a livelier critical dialogue on the role of scholars will be an internal priority. A collective dialogue is important as a minimal requirement. This, to avoid reproducing the most obvious problems with migration law: that only very few people manage to get through the individual examination and access the right to protection.

In hindsight, I can see how the different expectations of what law can do, and of academics’ role, and the frustrations these expectations led to, are also linked to other aspects such as the involved actors’ main occupations. Remembering the work, my approach was rather naive, seeing us all as activists. The logic in the asylum rights movement is more focused on meeting immediate needs, while researchers’ work is characterized by the law of slowness. With an incident such as the fire in the Moria refugee camp, for instance, or when a need arose for urgent litigation to stop a deportation, other concerns were left aside, for understandable reasons. However, our deadlines – for which I was responsible as the Commission’s research leader – did not take any such considerations into account.

At times, the above dilemmas could be productive: *First*, in that the knowledge we gained in legal aid sessions and advocacy work guided our circulation of relevant knowledge about the temporary law. *Second*, the different views on whether, how and when activist methods may be risky, may deepen conversations around strategic choices.

Listening to testimonies and sharing stories was important for the Commission’s mobilization work. By transforming experiences into explicit claims, such as on the blog and in the Asylum Archive, and having stories corroborated with reference to previous research, such as in the Shadow Directive, we could claim to provide an account that could be received as validated.

Further challenges in the Commission which I have not discussed in this article concerned who was invited to participate, who provided testimonies and who did not, and that the asylum seekers we worked with did not come forward in their full range of identities. Another problem was that some voices were more violently excluded from the public debate. Occasionally, with repeated encounters, through relationships and friendships, we were able to present ourselves to each other as whole people. This component is not something I have been able to address here but would be interesting to pursue in a future article.

## 8 Final words

Possibly, a deconstruction of the premises of migration law as a logical and reasonable restriction of people’s wish to move freely could be challenged, based on the work of the Asylum Commission. When times are less imperious and more progressive, the testimonies we collected and the documentation we created could be used in practical work towards a world order where membership does not necessarily go together with a residence permit. If it turns out that the law only confirms the repressive policies coming from above, it will become obvious that the system does not hold, and here also lies an opening for something new to emerge.

As I write these words, it is in the wake of valuable feedback from reviewers regarding, among other things, the impact of time on memory (one important text I have read is Sawyer and Osei-Kofi, 2020). Several legislative processes are now underway which aim to further discourage potential new groups of refugees from coming to Sweden, as well as implementing a “paradigm shift in the *view* of asylum immigration” (The Tidö Agreement, 2022, p. 29, my emphasis). One controversial proposal of particular concern in higher education institutions is that public officials, including university educators, should be obliged to report undocumented migrants whom they encounter in the course of their work to the border police (Lind et al., 2023). This has led to widespread criticism and an ongoing mobilization amongst university educators, especially those of us who work in the field of migration (Barinaga et al., 2023; FARR, 2023). If there had been a requirement to report undocumented people before the Asylum Commission was established, we would not have been able to carry out the project.

As time passes, the increasingly repressive policies, and yet another revisitation through slow reading of my diary notes, it becomes evident that my own perception of the Commission as well as my role in it, evolves. A lesson that emerges is that the activist Anna and the researcher Anna are two sides of the same coin and indeed a prerequisite for operating in politically sensitive areas where power relations are extremely unequal. Others’ expectations in the context of the Commission – the (external) legal positivist Swedish legal culture, and the (internal) goal to disrupt power and working with the law – remains complex and are not possible to fully grasp. Nor can we know what our engaged research might lead to in the long run. But we shall keep listening, writing, produce knowledge and circulate this is different ways. As I read in a later section of my own notes:

*Despite the unfeasibility of reaching those in power, we have kept on addressing both politicians and the wider public through* var*ious channels. Since the new legal changes came into force with the new Act [the permanent Aliens Act in 2021], asylum and immigration have been little discussed in mainstream media, and even less is asylum discussed as a fundamental right that states have committed to respect. The word ‘refugee’ has virtually disappeared from the public discourse, and other issues, often presented as being directly linked to immigration, have moved up the agenda. The legislative proposals currently under scrutiny include increasing the number of expulsions; demand-driven integration policies; a system of performance-based access to welfare rights; and severely restricted access to rights during the asylum process* (see the The Tidö Agreement, 2022).

Another realization that came to me is that the alliance building and political participation (Zemans, 1983) that took place within the Commission has continued in new constellations. It has become clear that when people come together and create communities struggling for a different future, the effects can never be predicted. This is also where the hope for a different future lies.

## Data availability statement

The data described in this study can be found online via the following link: asylkommissionen.se.

## Ethics statement

The studies involving humans were approved by ethical vetting board in Sweden. The studies were conducted in accordance with the local legislation and institutional requirements. Written informed consent for participation in this study was provided by the participants’ legal guardians/next of kin.

## Author contributions

AL: Investigation, Writing – original draft.
